# Diagnostic, prognostic and therapeutic implications of carbonic anhydrases in cancer

**DOI:** 10.1038/sj.bjc.6600936

**Published:** 2003-07-01

**Authors:** C P S Potter, A L Harris

**Affiliations:** 1 Cancer Research UKGrowth Factor Group, Weatherall Institute of Molecular Medicine, John Radcliffe Hospital, Oxford OX3 9DS, UK

**Keywords:** carbonic anhydrase, microenvironment; pH, hypoxia-inducible factor

## Abstract

The carbonic anhydrases (CAs) comprise a family of evolutionarily ancient enzymes found ubiquitously in nature. They have important roles in facilitating transport of carbon dioxide and protons in the intracellular space, across biological membranes and in the unstirred layers of the extracellular space. The tumour-associated isoenzymes, CAIX and CAXII, are expressed in a wide variety of malignancies and appear to be tightly regulated by microenvironmental hypoxia. CAIX expression is linked to poor prognosis in a number of human tumours, and may be a marker of aggressive malignant phenotype and a mechanism of progression. Inhibitors of CA may inhibit tumour growth and invasion, with consequent therapeutic potential.

The carbonic anhydrase (CA) family of zinc metalloenzymes is phylogenetically ancient, diverse in structure and its members are found in almost every living organism (Tripp *et al*, 2001). This short review aims to address recent advances in the association of CA with cancer biology, with particular attention to the novel tumour-associated CAs, IX and XII.

## FUNCTIONS OF CA


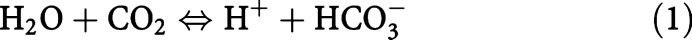


The above reaction (1) is ubiquitous in nature, involving the interchange of gaseous and ionic species crucial to a wide range of physiological and biochemical processes. Biological membranes form an effective barrier to the passive diffusion of bicarbonate and hydrogen ion, whereas carbon dioxide is highly membrane permeable, and it has been suggested that CA initially evolved to facilitate trans-cellular carbon dioxide transport rather than its more familiar role in respiratory gas exchange (Henry and Swenson, 2000). Certainly, at the single-cell level, carbon dioxide diffuses more rapidly in buffer solutions and across artificial membranes than would be expected from its diffusion coefficients, and this facilitated diffusion is abolished by the inhibition of CA activity (Geers and Gros, 2000).

**Figure 1:**
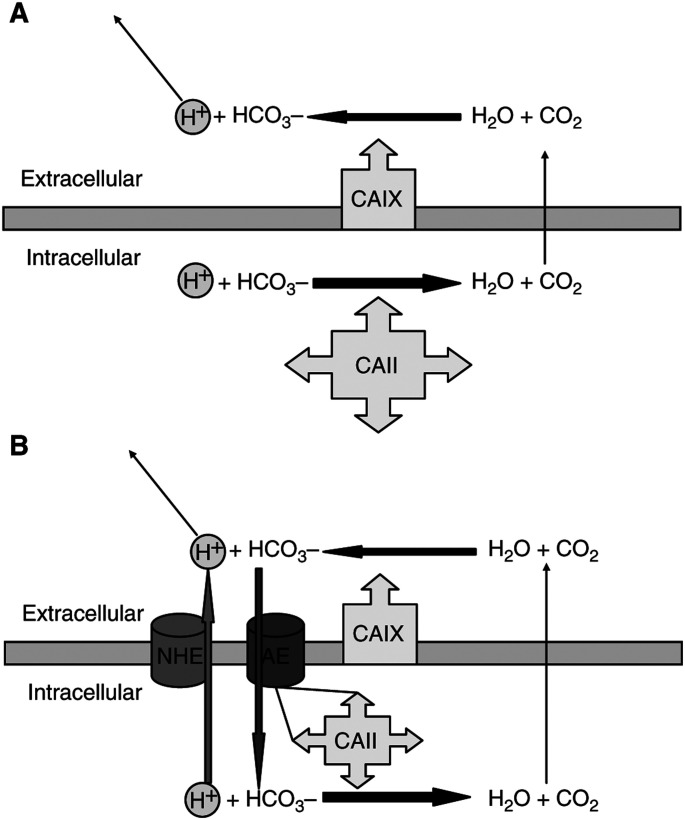
(**A**) Hydrogen ion produced by anaerobic metabolism within the cell must first be converted to carbon dioxide to facilitate diffusion across the lipid bilayer. This reaction with bicarbonate is catalysed by cytoplasmic CA. Once carbon dioxide has diffused into the extracellular space, CA activity in the boundary layer may regenerate protons, maintaining the carbon dioxide diffusion gradient. (**B**) The NHE is one method by which protons may be actively pumped from the cell. Alternatively, bicarbonate in the extracellular space (regenerated from carbon dioxide hydration with CA catalysis) may be imported into the cell via the chloride–bicarbonate AE. This bicarbonate may recombine with a proton in the cytoplasm to undertake further shuttling across the membrane. This recycling of bicarbonate with net proton extrusion is known as a Jacobs–Stewart cycle. The AE has recently been shown to bind CAII on its intracellular surface

Carbonic anhydrase may also confer directionality on carbon dioxide transport across membranes, maintaining high levels of the gas in solution on the upstream side of the membrane, and causing acidification of the downstream boundary layer thus maintaining the concentration gradient to drive diffusion (Figure 1A

).

In addition to facilitating passive diffusion, CAs may act in concert with membrane-associated ion transport systems such as the sodium–hydrogen exchanger (NHE) and chloride–bicarbonate anion exchanger (AE). Indeed, CAII is known to bind to the cytoplasmic tail of the band III AE in the erythrocyte, forming a metabolon, a physically associated complex of proteins in a sequential metabolic pathway (Sterling *et al*, 2001) (Figure 1B).

The three distinct CA families (*α*, *β* and *γ*) show no significant sequence identity and appear to have completely separate phylogenetic origins, a remarkable example of convergent evolution.

## THE *α*-CA FAMILY

There are 14 known members of this family, the only CAs to be found in mammalian cells. Eleven members express CA activity, whereas the three CA-related-polypeptides (CA-RPs VIII, XI and XIII) lack one or more of the critical histidine residues responsible for zinc ion binding at the active site. CAII is thought to be the most active enzyme found in nature, with a *K*_cat_ of around 10^6^ s^−1^, providing near-instantaneous equilibrium between the chemical species.

The family may be subdivided, on the basis of cellular localization, into cytosolic (CAs I, II, III, VII), membrane associated (CAs IV, IX, XII, XIV), mitochondrial (CAV) and secreted (CAVI). On the basis of intron : exon relations, the cytoplasmic and mitochondrial groups seem distinct from the trans-membrane and secreted enzymes. The highly heterogeneous distribution of the various isoenzymes within tissues, organs and cells suggests functionally distinct roles in processes as diverse as acid–base balance, gas exchange, ion transport, carbon fixation and mucosal protection.

Table 1

**Table 1:** Expression of cytoplasmic CAs in human tumours

**Tumour**	**CA expression pattern**
Central Nervous System	CAII staining maintained in astrocytomas, oligodendrocytomas and medulloblastomas. Staining appeared stronger in more malignant tumours(Parkkila et al., 1995)
Colorectal	CAsI and II expression reduced as differentiation lost, with reduced immunostaining correlating with malignant progression.CAI expression associated with reduced vascular invasion and good prognosis in colorectal tumours, CAII expression associated with good prognosis in rectal tumours(Bekku et al., 2000).
Lung	CAI and II expression reduced in squamous cell and adenocarcinoma(Chiang et al., 2002).
Haematological	CAI is a potential marker of erythroid differentiation in blast cells(Walloch et al., 1986) and CAII is found in a majority of acute leukaemias(Leppilampi et al., 2002).

Bekku S, Mochizuki H, Yamamoto T, Ueno H, Takayama E, Tadakuma T (2000) Expression of carbonic anhydrase I or II and correlation to clinical aspects of colorectal cancer. *Hepatogastroenterology*, **47**; 998–1001 Chiang WL, Chu SC, Yang SS, Li MC, Lai JC, Yang SF, Chiou HL, Hsieh YS (2002) The aberrant expression of cytosolic carbonic anhydrase and its clinical significance in human non-small cell lung cancer. *Cancer Lett* **188**; 199–205 Leppilampi M, Koistinen P, Savolainen ER, Hannuksela J, Parkkila AK, Niemela O, Pastorekova S, Pastorek J, Waheed A, Sly WS, Parkkila S, Rajaniemi H (2002) The expression of carbonic anhydrase II in hematological malignancies. *Clin Cancer Res* **8**; 2240–2245 Parkkila S, Parkkila AK, Juvonen T, Lehto VP, Rajaniemi H (1995) Immunohistochemical demonstration of the carbonic anhydrase isoenzymes I and II in pancreatic tumours. *Histochem J* **27**; 133–138 Walloch J, Frankel S, Hrisinko MA, Weil SC (1986) Carbonic anhydrase: a marker for the erythroid phenotype in acute nonlymphocytic leukemia. *Blood* **68**; 304–306

describes the expression patterns of the cytoplasmic CAs in human tumfours. It would appear that CAs I and II have some potential clinical utility as markers of differentiation for a number of cell types.

## NOVEL TUMOUR-ASSOCIATED TRANSMEMBRANE CAs

### CAIX

Interest in cancer-related CAs increased with the finding that the tumour-associated protein MN, discovered in HeLa cells cocultured with breast cancer cells, contained a CA domain very similar to that of CAIV (Pastorek *et al*, 1994). This 54/58 kDa *N*-glycosylated transmembrane protein also has an N-terminal region which shows significant homology to the keratan sulphate-binding domain of aggrecan (Opavsky *et al*, 1996), the major proteoglycan of articular cartilage, thought to be important in maintenance of tissue hydration. This pattern of a CA-related domain being found adjacent to a proteoglycan domain is also found in a number of other proteins, most notably the receptor protein tyrosine phosphatases (RPTPs *β* and *γ*) and the rat neural protein, phosphacan. The CA-like domain of RPTPβ is known to act as a ligand-binding site for the neuronal cell recognition molecule contactin (Peles *et al*, 1995), suggesting a role distinct from catalysis for this domain.

The MN gene thus appears to be chimeric in nature, arising from exon shuffling. Its sequence has been published (Opavsky *et al*, 1996), the original cDNA sequence corrected (Pastorek *et al*, 1994) and the sequences from tumours and normal tissue shown to be identical (Pastorekova *et al*, 1997). In 1996, the gene was renamed CA9 and its product has shown significant CA activity when expressed in COS cells (Sly, 2000), a truncated construct even showing equivalent activity to CAII (Wingo *et al*, 2001). Targeted disruption of CA9 gene expression in a murine model results in gastric glandular hyperplasia with proliferation of mucus-secreting pit cells, but otherwise normal development (Gut *et al*, 2002).

CA9 has been suggested to be a proto-oncogene on the basis of a number of observations:

1. Expression in HeLa cells is density dependent, CAIX expression increasing in confluent cultures (Zavada *et al*, 1993)
2. In HeLa/fibroblast hybrid cell lines, tumorigenicity in nude mice correlated with CA expression levels (Pastorek *et al*, 1994)
3. Transfection of NIH3T3 fibroblasts with the CA9 gene resulted in a transient transformation, with uncontrolled proliferation, growth in soft agar and morphological changes. After a few passages, the cells reverted to normal phenotype (Pastorek *et al*, 1994)
4. There is differential expression of CAIX between normal tissue and tumour specimens. Immunostaining and Northern blot techniques have revealed an extremely limited distribution in normal tissues, moderate expression being found in gastric mucosa, fetal lung and muscle. More sparse expression is found in small intestine, biliary tree and the male reproductive tract. Conversely, CAIX mRNA has been found in 50 of 87 malignant cell lines in one study (Ivanov *et al*, 2001)

The association with gastric hyperplasia in the mouse knockout suggests that any action may be tissue specific.

### CAXII

Originally patented in 1994 as a novel protein specific for lung cancer cells, CAXII is now recognised to be present in a wide variety of normal tissues and tumours (Tureci *et al*, 1998). The 39 kDa transmembrane protein shows a great deal of structural homology with CAIX, but lacks the proteoglycan domain. X-ray crystallography has revealed a dimeric structure, with a characteristic active site which may be susceptible to specific inhibitors (Whittington *et al*, 2001). Its expression in tissues with high absorptive capacities for water (colon, collecting duct, ascending loop of Henl) suggests a role in normal tissue physiology distinct to that of CAIX (Parkkila *et al*, 2000a).

## CONTROL OF CAIX AND CAXII EXPRESSION

A number of microenvironmental factors were initially shown to induce CAIX expression *in vitro* – notably confluent growth and suspension culture (Lieskovska *et al*, 1999).

Both CAs were shown by our group to be induced by hypoxia in a wide range of malignant cells *in vitro* including bladder, breast, cervical and lung cancer lines (Wykoff *et al*, 2000). The von-Hippel–Lindau tumour suppressor gene appears to play a critical role in this process, clear cell renal carcinoma cell lines with mutant VHL expressing both CAIX and CAXII constitutively. Both RNA differential display (Ivanov *et al*, 1998) and RNAse protection assay (Wykoff *et al*, 2000) have shown that reintroduction of the wild-type VHL gene into the same cell lines results in downregulation of these CAs in normoxia, with a restoration of the hypoxic response. We found a binding site for hypoxia inducible factor 1*α* (HIF-1*α*), the hypoxia response element (HRE) in the CA9 promoter and demonstrated that hypoxic induction is absent in cell lines defective for the HIF pathway, but may be restored by transfection of human HIF-1*α*. Mutations made within the core of the HRE also abrogate the hypoxic response, confirming the pivotal role of the HIF pathway (Wykoff *et al*, 2000). The strong inducibility in hypoxia that is conferred by the minimal CA9 promoter may be of use in targeting gene therapy vectors to areas of tumour hypoxia (Dachs *et al*, 1997). Other factors may be of significance in producing the strong upregulation of CAIX in cancer, since p53 mutation modulates expression, the promoter is less methylated in cancer (Cho *et al*, 2001) and contains binding sites for activator protein 1 and specificity protein transcription factors 1 and 3 (Kaluzova *et al*, 2001).

Other HIF target genes include glucose transporters, glycolytic enzymes and angiogenic growth factors such as VEGF, all essential for survival in a hostile, hypoxic environment. The tumour-associated CAs may play a role in maintenance of an acidic extracellular pH, an important element of the malignant phenotype (Ivanov *et al*, 2001). Although lactate produced by glycolysis under hypoxic conditions is a significant contributor to acidic extracellular pH, there is also a substantial contribution from carbonic acid (Griffiths *et al*, 2001).

## CAIX AS A MARKER OF HYPOXIA

Hypoxic tumours are known to have a relatively poor prognosis, independent of the modality of treatment used (Vaupel, 1997). Current methods of measuring tumour oxygenation are either invasive (Eppendorf microelectrode) or require administration of chemical agents (e.g. pimonidazole). There are obvious benefits of an endogenous hypoxia biomarker that is nondiffusible (unlike VEGF) and easily processed in paraffin sections for staining. Thus, it is important to demonstrate that the *in vitro* findings with regard to CA induction by hypoxia can be confirmed *in vivo*.

In a multicellular spheroid model, CAIX immunostaining was more marked on the plasma membrane of cells from the innermost layers, with a distribution similar to that of pimonidazole. Similarly, human glioma xenografts grown in immunodeficient mice show more CAIX-staining in poorly perfused and hypoxic areas. The CAIX-stained cells remained viable when plated out, and were shown to be more radioresistant than unstained cells (Olive *et al*, 2001).

*In vivo* studies in human tumours have confirmed these findings, revealing a predominantly perinecrotic staining pattern. In squamous cancers of the head and neck, we observed a gradient of CA9 expression with highest levels adjacent to frank necrosis and considerable overlap with pimonidazole staining (Beasley *et al*, 2001). Biopsies of invasive cervical carcinomas also showed a good correlation between staining for the two hypoxia markers, CA9 staining being more extensive in almost all cases (Olive *et al*, 2001).

CD34 staining of the microvasculature reveals a median distance of 80 *μ*m between a vessel and CAIX expression in squamous head and neck tumours, which corresponds to a tissue oxygen tension of around 1%. This corresponds to the level at which HIF-1*α* and its target genes are induced. In this study, CAIX staining was also significantly related to levels of tumour necrosis (Beasley *et al*, 2001).

Papillary renal tumours, breast, bladder and ovarian cancers show a similar perinecrotic CAIX distribution. Conversely, renal clear cell carcinomas showed a uniform staining pattern irrespective of areas of hypoxia and necrosis, suggesting a constitutively upregulated HIF pathway in these tumours (Wykoff *et al*, 2000).

Definitive measurement of tumour hypoxia by Eppendorf microelectrode has confirmed that there is a significant positive correlation between the hypoxic fraction of advanced cervical carcinomas and the extent of CAIX immunostaining (Loncaster *et al*, 2001). Whether the above findings will translate to significant prognostic information in a prospective trial remains to be determined, but accumulating evidence from a number of clinical studies (see Table 2

**Table 2:** Expression of membrane-associated CAs in human tumours

**Tumour**	**CA expression pattern**
Renal	CAIX expressed in renal cell carcinomas and Von Hippel–Lindau-associated tumours (Wykoff *et al*, 2000), but not normal tissue or other renal cancers. CAIX is identical to G250 tumour-associated antigen, therapeutic target in clinical trials (Divgi *et al*, 1998). PCR of peripheral blood may detect CAIX-positive renal cancer cells in circulation, potential diagnostic biomarker (de la Taille *et al*, 2000). CAXII expression found in most oncocytomas and renal cell carcinomas (Parkkila *et al*, 2000)
Cervical	CAIX expressed in majority cervical squamous cell carcinomas, significant independent negative predictor of survival (Loncaster *et al*, (2001). Expression in smear parallels that of tissue biopsies and correlates with clinically significant disease at biopsy (Liao and Stanbridge 2000).
Squanous carcinoma of the head and neck	CAIX Immunostaining perinecrotic and associated with advanced disease (Beasley *et al*, 2001), poor radiosensitivity and short survival (Koukourakis *et al*, 2001).
Lung	Fifty percent squamous carcinoma and 16% adenocarcinomas express CAIX (O'Byrne *et al*, 2001), absent in dysplastic (Vermylen *et al*, 1999) and normaltissues.Expression increases with advanced stage disease and is a significant adverse prognostic factor (Giatromanolaki *et al*, 2001).
Breast	Fifty percent ductal carcinoma-*in situ* and 29% invasive carcinomas positive for CAIX immunostaining, associated with necrosis, high-grade and poor prognosis. (Chia *et al*, 2001) CAXII staining found in 89% normal breast samples, 84%DCIS and 71% invasive carcinomas, associated with low-grade and good prognosis. (Wykoff *et al*,2001)
Colorectal	CAIX exspression parallels cellular proliferation and increases with reduced cellular differentiation being more pronounced in frank adenocarcinoma than dysplasia or adenomatous disease (Saarnio *et al*, 1998). CAXII expression is also increased in adenocarcinoma(Kivela *et al*, 2000a).
Oesophageal	CAIX expression reduced in adenocarcinoma compared to dysplasia. (Turner *et al*, 1997)
Gastric	CAIX expression reduced in gastric carcinoma (Pastorekova *et al*, 1997)
Biliary tree	CAIX expression increased in hyperplastic, dysplastic (Kivela *et al*, 2000b) and malignant ductal epithelium (Saarnio *et al*, 2001).
Bladder	CAIX expression increased in superficial tumours. (Turner *et al*, 2002)

Beasley NJ, Wykoff CC, Watson PH, Leek R, Turley H, Gatter K, Pastorek J, Cox GJ, Ratcliffe P, Harris AL (2001) Carbonic anhydrase IX, an endogenous hypoxia marker, expression in head and neck squamous cell carcinoma and its relationship to hypoxia, necrosis, and microvessel density. *Cancer Res* **61**; 5262–5267 Chia SK, Wykoff CC, Watson PH, Han C, Leek RD, Pastorek J, Gatter KC, Ratcliffe P, Harris AL (2001) Prognostic significance of a novel hypoxia-regulated marker, carbonic anhydrase ix, in invasive breast carcinoma. *J Clin Oncol* **19**; 3660–3668 de la Taille A, Buttyan R, Katz AE, McKiernan J, Burchardt M, Burchardt T, Chopin DK, Sawczuk IS (2000) Biomarkers of renal cell carcinoma. Past and future considerations. *Urol Oncol* **5**; 139–148 Divgi CR, Bander NH, Scott AM, O'Donoghue JA, Sgouros G, Welt S, Finn RD, Morrissey F, Capitelli P, Williams JM, Deland D, Nakhre A, Oosterwijk E, Gulec S, Graham MC, Larson SM, Old LJ (1998) Phase I/II radioimmunotherapy trial with iodine-131-labeled monoclonal antibody G250 in metastatic renal cell carcinoma. *Clin Cancer Res* **4**; 2729–2739 Giatromanolaki A, Koukourakis MI, Sivridis E, Pastorek J, Wykoff CC, Gatter KC, Harris AL (2001) Expression of hypoxia-inducible carbonic anhydrase-9 relates to angiogenic pathways and independently to poor outcome in non-small cell lung cancer. *Cancer Res* **1**; 7992–7998 Kivela A, Parkkila S, Saarnio J, Karttunen TJ, Kivela J, Parkkila AK, Waheed A, Sly WS, Grubb JH, Shah G, Tureci O, Rajaniemi H (2000a) Expression of a novel transmembrane carbonic anhydrase isozyme XII in normal human gut and colorectal tumors. *Am J Pathol* **156**; 577–584 Kivela AJ, Parkkila S, Saarnio J, Karttunen TJ, Kivela J, Parkkila AK, Pastorekova S, Pastorek J, Waheed A, Sly WS, Rajaniemi H (2000b) Expression of transmembrane carbonic anhydrase isoenzymes IX and XII in normal human pancreas and pancreatic tumours. *Histochem Cell Biol* **114**; 197–204 Koukourakis MI, Giatromanolaki A, Sivridis E, Simopoulos K, Pastorek J, Wykoff CC, Gatter KC, Harris AL (2001) Hypoxia-regulated carbonic anhydrase-9 (CA9) relates to poor vascularization and resistance of squamous cell head and neck cancer to chemoradiotherapy. *Clin Cancer Res* **7**; 3399–3403 Liao SY, Stanbridge EJ (2000) Expression of MN/CA9 protein in Papanicolaou smears containing atypical glandular cells of undetermined significance is a diagnostic biomarker of cervical dysplasia and neoplasia. *Cancer* **88**; 1108–1121 Loncaster JA, Harris AL, Davidson SE, Logue JP, Hunter RD, Wycoff CC, Pastorek J, Ratcliffe PJ, Stratford IJ, West CM (2001) Carbonic anhydrase (CA IX) expression, a potential new intrinsic marker of hypoxia: correlations with tumor oxygen measurements and prognosis in locally advanced carcinoma of the cervix. *Cancer Res* **61**; 6394–6399 O'Byrne KJ, Cox G, Swinson D, Richardson D, Edwards JG, Lolljee J, Andi A, Koukourakis MI, Giatromanolaki A, Gatter K, Harris AL, Waller D, Jones JL (2001) Towards a biological staging model for operable non-small cell lung cancer. *Lung Cancer* **34**; S83–S89 Parkkila S, Parkkila AK, Saarnio J, Kivela J, Karttunen TJ, Kaunisto K, Waheed A, Sly WS, Tureci O, Virtanen I, Rajaniemi H (2000) Expression of the membrane-associated carbonic anhydrase isozyme XII in the human kidney and renal tumors. *J Histochem Cytochem* **48**; 1601–1608 Pastorekova S, Parkkila S, Parkkila AK, Opavsky R, Zelnik V, Saarnio J, Pastorek J (1997) Carbonic anhydrase IX, MN/CA IX: analysis of stomach complementary DNA sequence and expression in human and rat alimentary tracts. *Gastroenterology* **112**; 398–408 Saarnio J, Parkkila S, Parkkila AK, Haukipuro K, Pastorekova S, Pastorek J, Kairaluoma MI, Karttunen TJ (1998) Immunohistochemical study of colorectal tumors for expression of a novel transmembrane carbonic anhydrase, MN/CA IX, with potential value as a marker of cell proliferation. *Am J Pathol* **153**; 279–285 Saarnio J, Parkkila S, Parkkila AK, Pastorekova S, Haukipuro K, Pastorek J, Juvonen T, Karttunen TJ (2001) Transmembrane carbonic anhydrase, MN/CA IX, is a potential biomarker for biliary tumours. *J Hepatol* **35**; 643–649 Turner JR, Odze RD, Crum CP, Resnick MB (1997) MN antigen expression in normal, preneoplastic, and neoplastic esophagus: a clinicopathological study of a new cancer-associated biomarker. *Hum Pathol* **28**; 740–744 Turner KJ, Crew JP, Wykoff CC, Watson PH, Poulsom R, Pastorek J, Ratcliffe PJ, Cranston D, Harris AL (2002) The hypoxia-inducible genes VEGF and CA9 are differentially regulated in superficial vs invasive bladder cancer. *Br J Cancer* **86**; 1276–1282 Vermylen P, Roufosse C, Burny A, Verhest A, Bosschaerts T, Pastorekova S, Ninane V, Sculier JP (1999) Carbonic anhydrase IX antigen differentiates between preneoplastic malignant lesions in non-small cell lung carcinoma. *Eur Respir J* **14**; 806–811 Wykoff CC, Beasley N, Watson PH, Campo L, Chia SK, English,R, Pastorek J, Sly WS, Ratcliffe P, Harris AL (2001) Expression of the hypoxia-inducible and tumor-associated carbonic anhydrases in ductal carcinoma *in situ* of the breast. *Am J Pathol* **158**; 1011–1019 Wykoff CC, Beasley NJ, Watson PH, Turner KJ, Pastorek J, Sibtain A, Wilson GD, Turley H, Talks KL, Maxwell PH, Pugh CW, Ratcliffe PJ, Harris AL (2000) Hypoxia-inducible expression of tumor-associated carbonic anhydrases. *Cancer Res* **60**; 7075–7083

) suggests that this may indeed be the case.

## CARBONIC ANHYDRASE AS A TARGET FOR THERAPY

Further evidence of the importance of the CAs in the oncogenic process comes from the use of CA inhibitors, most notably the heterocyclic and aromatic sulphonamides of which acetazolamide is the most prominent.

In bicarbonate-free medium, sulphonamides were effective in reducing growth rates of lymphoma cell lines according to their order of potency in CA inhibition (Chegwidden *et al*, 2000). The growth inhibition was reversed by supplementing the medium with nucleotide precursors, suggesting that these may be depleted most readily by the high metabolic flux of the transformed cell in a low bicarbonate environment. The critical step may involve carbamoyl synthetase II, a cytosolic enzyme that utilises bicarbonate for the first step in pyrimidine nucleotide biosynthesis.

Many novel sulphonamide derivatives have been shown to have growth-inhibitory effects on a variety of malignant cell lines *in vitro*, often at concentrations in the nanomolar range (Supuran *et al*, 2001). However, some sulphonamides without any CA-inhibitory activity also show growth-inhibitory effects, possibly because of inhibition of tubulin polymerisation. There is also potentially a degree of crossover with inhibition of other zinc metalloenzymes, notably the matrix metalloproteinases.

Acetazolamide has been shown to reduce invasiveness of four RCC cell lines *in vitro* by 18–74%, although the only cell line shown to express CAIX was also the least affected (Parkkila *et al*, 2000b). The concentrations used were orders of magnitude greater than that needed to inhibit CA.

Invasiveness is known to increase in cells cultured in low pH medium, and it is possible that the presence of CA activity may promote this acidification of the extracellular space, with concomitant activation of enzymes required for matrix degradation (Martinez-Zaguilan *et al*, 1996).

Acetazolamide has shown antitumour properties in a murine fibrosarcoma model, producing significant growth delays when used as a single agent, and additive growth delays in combination with a number of chemotherapeutic agents (Teicher *et al*, 1993).

In the clinical setting, the differential expression of CAIX in renal cancers has provided a target for radioimmunotherapy, antibody-mediated gene transfer and vaccination strategies (Tso *et al*, 2001), and phase I/II trial results with iodine-131 labelled murine monoclonal antibodies have been performed with some antitumour activity (Divgi *et al*, 1998). The development of a humanised monoclonal antibody is awaited to allow repeated cycles of treatment.

Many chemotherapeutic drugs are weak acids or bases, their pK_a_'s being in the physiological range. As most of these drugs enter the cell by passive diffusion and the plasma membrane is relatively impermeable to ionised species, slight differences in pH on either side of the membrane may result in dramatic alterations in the distribution of the drug due to ion trapping. In areas of tumour distant from the vasculature, extracellular pH may be expected to drop, whereas the intracellular pH remains relatively constant, increasing the intra- to extracellular pH gradient and favouring the uptake of weak acid drugs. Many clinically useful chemotherapeutic drugs are weak bases (e.g. doxorubicin, bleomycin, mitoxantrone), whose uptake in animal models may be enhanced by bicarbonate administration, reducing the extracellular acidity of tumours (Raghunand and Gillies, 2001). Whether CA inhibitors may be effective in reducing this tumour acidity and consequently have a role in combination chemotherapy remains to be demonstrated.

## CONCLUSIONS

The intracellular pH in solid tumours remains close to the physiological value despite the relative acidity of the interstitial space under these circumstances, suggesting that malignant cells extrude protons more avidly than their untransformed counterparts. Studies on retinal pigmented epithelia have demonstrated a drop in intracellular pH following selective inhibition of extracellular CA by a membrane-impermeant agent, associated with reduced activity of the NHE (Wu *et al*, 1998). Similar effects have been seen in muscle, with reduced proton and lactate export when extracellular CA is inhibited (Geers and Gros, 2000). Acetazolamide will also inhibit activity of the AE,1 a protein functionally and physically linked to CAII and critical to maintaining cytoplasmic pH (Sterling *et al*, 2001). Both NHE and AE expression is also modulated by the HIF pathway (Karumanchi *et al*, 2001).

Extracellular CA would appear to be in an ideal position to convert carbon dioxide diffused across the plasma membrane to protons and bicarbonate, the latter being transported back into the cell by the AE, forming a Jacobs–Stewart cycle (Figure 1B), with net extrusion of protons.

The consequent acidification of the extracellular space would in addition be permissive for invasion, and have an adverse effect on cell-mediated immunity. Hypoxia-induced apoptosis may also be dependent on a drop in extracellular pH (Schmaltz *et al*, 1998) providing selective pressure for apoptosis-resistant malignant clones. The acidic microenvironment of tumours has long been assumed to be because of excess lactate production by anaerobic metabolism, but cells deficient in lactate production produce equally acidic interstiae (Griffiths *et al*, 2001) suggesting the importance of a distinct pathway for acidification such as that outlined above.

In addition to its role in transmembrane solute transport, extracellular CA may be important in facilitated diffusion of protons/carbon dioxide in the unstirred layers of the extracellular space. Rapid interconversion of the two species in the presence of mobile buffers will result in more rapid diffusion down the concentration gradient to better perfused areas, dissipating pH gradients that may otherwise preclude further tumour growth (Griffiths *et al*, 2001).

The presence of a proteoglycan/cell adhesion domain on the CAIX protein is highly suggestive of a role in cell–cell or cell–matrix interaction. The majority of CAIX expression in normal tissues would appear to be on the basolateral plasma membrane, and cell attachment to CAIX *in vitro* can be inhibited by monoclonal antibodies directed to this domain (Zavada *et al*, 2000). The cytoplasmic tail has not yet been shown to partake in a signal transduction pathway (unlike the RPTPs), but this has not been fully investigated.

As more is learnt about the mechanism of action of the tumour-associated CAs, it is hoped that they may make the transition from biomarkers of hypoxia and differentiation to essential tumour enzymes and therapeutic targets. The development of specific inhibitors for CAs IX and XII may facilitate this process, leading to a greater understanding of the role in tumour biology of these recently discovered yet phylogenetically ancient enzymes.
